# How inversion variants can shape neural circuitry: Insights from the three-morph mating tactics of ruffs

**DOI:** 10.3389/fphys.2022.1011629

**Published:** 2022-10-25

**Authors:** Jasmine L. Loveland, Lina M. Giraldo-Deck, Aubrey M. Kelly

**Affiliations:** ^1^ Department of Cognitive and Behavioral Biology, University of Vienna, Vienna, Austria; ^2^ Behavioural Genetics and Evolutionary Ecology, Max Planck Institute for Biological Intelligence (in Foundation), Seewiesen, Germany; ^3^ Department of Psychology, Emory University, Atlanta, GA, United States

**Keywords:** HSD17B2, supergene, chromosome inversion, aromatase, vasotocin, alternative reproductive tactics, *Calidris pugnax*, testosterone

## Abstract

Behavior polymorphisms underlying alternative mating tactics can evolve due to genetic inversions, especially when inversions capture sets of genes involved in hormonal regulation. In the three-morph system of the ruff (*Calidris pugnax*), two alternative morphs (Satellites and Faeders) with distinct behaviors and low circulating testosterone are genetically determined by an inverted region on an autosomal chromosome. Here, we discuss recent findings on the ruff and present novel insights into how an inversion that poses drastic constraints on testosterone production might lead to morph-specific differences in brain areas that regulate social behavior. A gene responsible for converting testosterone to androstenedione (*HSD17B2*) is located inside the inverted region and is a promising candidate. We identify a single missense mutation in the *HSD17B2* gene of inverted alleles that is responsible for a 350–500% increase in testosterone to androstenedione conversion, when mutated in the human HSD17B2 protein. We discuss new evidence of morph differences in neural *HSD17B2* expression in embryos and circulating androgens in sexually-immature juveniles. We suggest processes that shape morph differences in behavior likely begin early in ontogeny. We propose that the organization of behaviorally relevant neuron cell types that are canonically sexually dimorphic, such as subpopulations of aromatase and vasotocin neurons, should be particularly affected due to the life-long condition of low circulating testosterone in inversion morphs. We further emphasize how HSD17B2 catalytic activity extends beyond androgens, and includes estradiol oxidation into estrone and progesterone synthesis. Lastly, we underscore dimerization of HSD17B2 as an additional layer of complexity that merits consideration.

## Introduction

### The ruff: Behavior, hormones, and genetics

**FIGURE 1:**
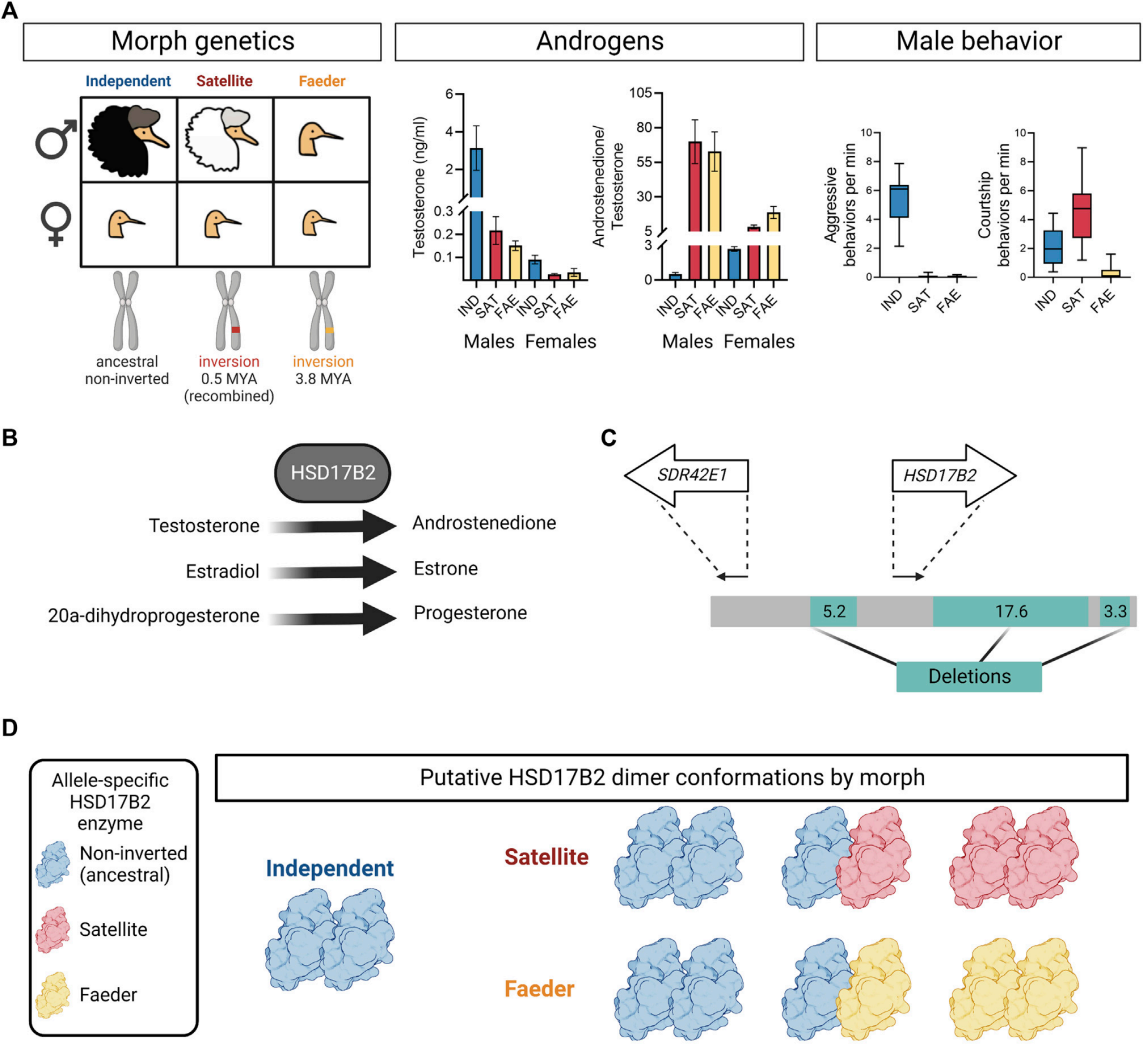
Overview of ruff morph genetics, testosterone, behavior and HSD17B2 features. **(A)** Illustration of morph profiles with corresponding genetics, circulating testosterone levels, androstenedione to testosterone ratio, and male behavior during the breeding season. In males, Independents and Satellites are ornamented, while Faeders are not. Satellites and Faeders are carriers of distinct inversion haplotypes on chromosome 11, thus present in both sexes. The Faeder inversion occurred 3.8 million years ago (MYA) and the Satellite inversion appeared ∼0.5 MYA due to a recombination event between Independent and Faeder alleles. In both sexes, inversion morphs have lower circulating levels of testosterone and higher androstenedione to testosterone ratio, compared to Independent counterparts. Error bars are ± SEM. The *y*-axis breaks were introduced to provide informative detail. Boxplots show only Independent males aggressively defend territories and both Independents and Satellites actively court females, often with co-displays. Independent, IND; Satellite, SAT; Faeder, FAE. Plotted sample sizes in order Independent, Satellite, Faeder for testosterone in males: 17, 9, 9; females: 7, 5, 3; for androstenedione to testosterone ratio in males: 10, 7, 7; females: 5, 3, 3 [Data from (Loveland et al., 2021a) and females were processed in parallel]. **(B)** The HSD17B2 enzyme catalyzes with high affinity testosterone to androstenedione, estradiol to estrone and also with lower affinity, 20-alpha-dihydroprogesterone to progesterone. **(C)** Illustration of deletions (green) surrounding *HSD17B2* and *SDR42E1* genes inside inversion haplotypes. Both Faeder and Satellite haplotypes have these deletions. Arrows depict relative locations of *SDR42E1* and *HSD17B2* genes oriented in their direction of transcription. Numbers denote deletion sizes in kilobases. Redrawn based on (Lamichhaney et al., 2016). **(D)** Putative HSD17B2 dimer conformations by morph. As inversion heterozygotes, Satellites and Faeders are expected to express two HSD17B2 alleles: non-inverted (blue) and inversion (red, Satellite; yellow, Faeder) alleles. Therefore, if HSD17B2 acts in a dimer conformation, then Satellites and Faeders may have up to three possible dimer assemblies due to their heterozygosity, whereas Independents have only one possible homodimer. Solely for illustration purposes PDB ID: 6MNE was used in lieu of a crystal structure for HSD17B2

Ruffs (*Calidris pugnax*) have long fascinated ornithologists because of their ornamental breeding plumage and lekking behavior, though the genetic basis for their unique mating system was discovered only recently (Küpper et al., 2016; Lamichhaney et al., 2016). Their mating system consists of three genetically determined morphs called Independents, Satellites and Faeders, that have striking differences in behavioral repertoires, hormonal profiles, nuptial plumage and body size (Hogan-Warburg, 1966; van Rhijn, 1973; Küpper et al., 2016; Lamichhaney et al., 2016; Loveland et al., 2021a) (Figure 1A).

Independent males aggressively defend mating courts on leks, display courtship behaviors and have dark colored ruffs. The behavior of Independent males is so intense that in some languages the species name is simply “fighter” or “walking fighter” (*combatiente*, Spanish; *Kampfläufer*, German). In contrast, Satellite males are not aggressive, co-display courtship behaviors with Independent males and have mostly white colored ruffs. Faeder males do not display courtship or aggressive behaviors, lack ruff feathers and are the smallest of the three morphs (Figure 1A) (Jukema and Piersma, 2006; Lank et al., 2013). Given their cryptic appearance, Faeders pass as female mimics and rely on a strictly sneaker strategy to attain matings.

The genetic polymorphism underlying the alternative mating strategies in ruffs is controlled by a 4.5 Mb inversion, also called a supergene, that contains approximately 125 genes (Küpper et al., 2016; Lamichhaney et al., 2016). Independents are the ancestral morph, whereas Satellites and Faeders are both inversion carriers, however, of distinct inversion haplotypes. Inversion haplotypes are dominant, homozygous lethal and contain several genes associated with sex steroid metabolism (Küpper et al., 2016; Lamichhaney et al., 2016; Loveland et al., 2021b). The Faeder inversion appeared first, 3.8 million years ago and the Satellite inversion arose by the recombination of Independent and Faeder alleles 500,000 years ago (Küpper et al., 2016; Lamichhaney et al., 2016) (Figure 1A). For simplicity, we will use “inversion morphs” to collectively refer to Satellites and Faeders, even though their inversion haplotypes are not identical.

Hormonally, adult Satellite and Faeder males have low levels of testosterone, but high levels of androstenedione, compared to Independents (Küpper et al., 2016; Loveland et al., 2021a) (Figure 1A). While the overt behavioral differences among male morphs are likely heavily influenced by differences in their capacity to produce testosterone (Loveland et al., 2021a), when and how this feature shapes their morph-specific behavioral repertoires is unknown. Here we will highlight key findings and ongoing work that informs future research aimed at mechanistic explanations of how inversion haplotypes facilitate the display of male morph-specific behaviors in the ruff.

### The *HSD17B2* gene

Since the discovery of the genetic basis for the alternative ruff morphs, the *HSD17B2* gene, located inside the inverted region, has been considered a key candidate to explain circulating androgenic differences between Independent and inversion morph males. *HSD17B2* encodes the hydroxysteroid 17-beta dehydrogenase two enzyme, which has been well characterized in humans and rodent models showing it converts with high affinity testosterone to androstenedione, estradiol to estrone, and with lower affinity 20-alpha-dihydroprogesterone to progesterone (Figure 1B) (Wu et al., 1993).

**FIGURE 2:**
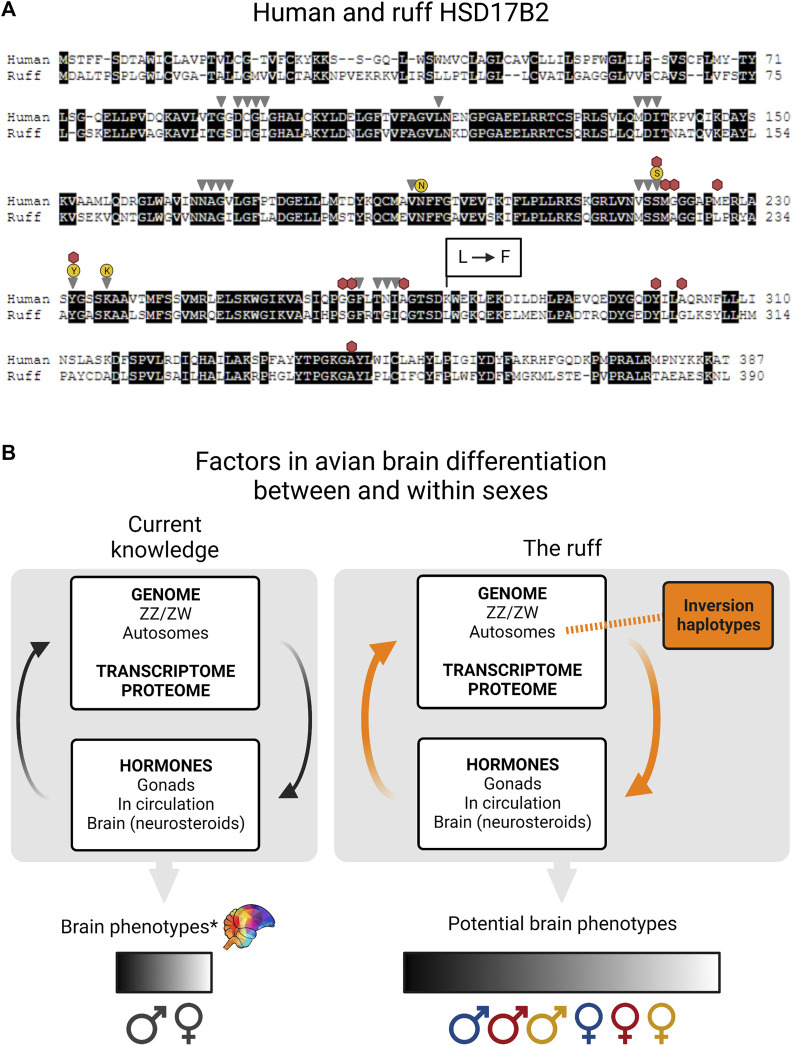
Missense mutation in ruff HSD17B2 and factors in avian brain differentiation. **(A)** Pairwise alignment of human and ruff HSD17B2 proteins (accession numbers NP_002144.1, XP_014797711.1) (BLOSUM45 matrix, internal gap penalty = -4) with identical residues in a black background). The 37 residues belonging to the HSD17beta type 2 conserved domain are indicated for NADP binding (gray triangles), steroid binding (red hexagons), catalytic tetrad (yellow circles). At ruff position 279, Satellite and Faeder alleles have the missense mutation that changes leucine to phenylalanine, indicated by L → F. Amino acid changes at this same position in the human protein increase catalytic activity for testosterone to androstenedione up to 5-fold compared to the non-mutated version (Sager et al., 2021). **(B)** The current knowledge of avian brain differentiation between and within the sexes relies on a series of complex interactions and here we show a simplified illustration of key factors: the genome, transcriptome, proteome and hormones. Sex chromosomes express specific genes that help determine gonadal sex, which in turn provides a hormonal environment from the synthesis of sex steroids from gonadal tissues. Sex hormones have a major effect on sexual differentiation. Once bound to receptors (i.e. androgen and estrogen receptors) these will then act as transcription factors and regulate the expression of target genes throughout the entire genome. Autosomes allow the expression of receptor genes to sense hormones and enzymes that can make hormones, including inside brain cells (neurosteroids). e.g. by aromatizing circulating testosterone into estradiol. All of these processes contribute to variation in brain phenotypes within and between sexes. We acknowledge that for any sex difference in a brain trait there can be overlap between the sexes, denoted by the spectrum (*) Even so, this is oversimplified and does not fully take fully into account brain mosaicism [reviewed in (Joel, 2011, 2021)]. In ruffs, inversion haplotypes (orange box) form part of the genome and have a direct impact on genetic architecture. The expression of inversion allele(s) results in dramatically reduced testosterone levels (and perhaps also reduced estradiol levels). By producing a reduction in the amount of circulating sex hormones, the inversion modifies the typical influence of sex chromosomes on brain sexual differentiation (orange arrows). We predict that morph variation will add to variation between the sexes, but this will be constrained by interactions between hormones and gene expression of each morph + sex combination. Independents (blue), Satellites (red), Faeders (yellow).

HSD17B2 enzyme function could have become different between Independents and inversion morphs through mutations in regulatory and/or coding regions. There are three major deletions surrounding the *HSD17B2* gene that could potentially lead to widespread or tissue-specific expression changes (Figure 1C) (Küpper et al., 2016; Lamichhaney et al., 2016). Both Satellites and Faeders have these deletions in their respective inversions. Ruff and human HSD17B2 share 54% and 67% sequence identity across the entire protein and ‘HSD17beta type 2 classical SDR domain’ sequences, respectively (Altschul et al., 1997). We confirmed that the ruff HSD17B2 possesses all functional domains described for the human HSD17B2 enzyme comprising 37 amino acid sites identified with the conserved domains search tool (Marchler-Bauer and Bryant, 2004; Lu et al., 2020) (Figure 2A).

While no crystal structure exists for any HSD17B2 enzyme in the Protein Database (Berman et al., 2000) recent *in silico* three-dimensional modeling, site-directed mutagenesis and *in vivo* functional validation experiments show that mutations to Lys275 in human HSD17B2 confer up to a 5-fold increase in the testosterone to androstenedione conversion rate, and approximately a 2-fold increase in the estradiol to estrone conversion rate (Sager et al., 2021). Based on the pairwise alignment of human and ruff HSD17B2 proteins, the corresponding position is mutated from Leu279Phe in both Satellite and Faeder alleles relative to the non-inverted allele (Figure 2A). We propose that this mutation could harbor profound explanatory power for the increased circulating androstenedione, and low testosterone, in inversion morphs. As such, it undoubtedly warrants further investigation to validate its role in modifying catalytic activity. This adds to our previous note on another missense mutation occurring near positions that form the ‘NSYK’ catalytic tetrad of this enzyme (Loveland et al., 2021b).

While we initially reported a lack of testicular *HSD17B2* mRNA expression differences among morphs (Loveland et al., 2021b), in our most recent work we show there are clear morph differences in *HSD17B2* mRNA expression in the brains of embryos, as early as day 14 of incubation (Giraldo-Deck, 2022). Interestingly, these morph differences, with higher expression in inversion morphs compared to Independents, are present in both sexes and occur in areas containing nodes of the social behavior network (Newman, 1999; Goodson, 2005). The detection of *HSD17B2* expression differences at such an early stage strongly suggests that in addition to low circulating testosterone, the presence of the inversion can also lead to changes in brain estradiol levels. This is because inversion morphs must experience atypical levels of estradiol in the brain due to 1) less aromatizable testosterone and 2) greater conversion of estradiol into estrone by HSD17B2. As we move forward, it will be important to maintain an integrative interpretation of results, keeping in mind that inversion effects occur in both sexes and that even in females this could have effects on behaviorally relevant cell types.

A key issue that has yet to be acknowledged is that the ruff HSD17B2 likely catalyzes in a homodimer conformation adding an entire new layer of complexity to how we speculate HSD17B2 function might differ among morphs. The human HSD17B2 has been shown to function unequivocally as a homodimer in solution (Lu et al., 2002). As dimerization is a requirement for function, then that means that HSD17B2 dimers in Satellites and Faeders should always be composed of three possible combinations of HSD17B2 enzymes (given expression of variants from non-inverted and inverted alleles) (Figure 1D). Thus, the new dimer conformations may confer a different catalytic activity compared to the homodimer composed of only HSD17B2 expressed from the non-inverted allele.

### Inversion haplotypes likely shape morph-specific neural organization

Foundational studies by Lank and others provided valuable insights and direction for inquiries into the neurobiological basis of behavioral differences in the ruff. In a key study, the authors reported that adult females implanted with testosterone displayed male behaviors and grew nuptial plumage that were typical to their corresponding male morph counterpart (Lank et al., 1999). This suggests that in the ruff, as is the case for several other bird species, broadly speaking, males are likely the “default” sex and females undergo a de-masculinization step by exposure to estrogens as embryos (Adkins-Regan, 1987). Considering the facts of differential *HSD17B2* gene expression in embryo brains and that morph differences in circulating androgens are detectable as early as the juvenile stage (Giraldo-Deck, 2022), we believe morph differences in neural organization may begin to take shape early in ontogeny. The behavioral effects of the inversion likely come from its ability to reduce not only circulating levels of testosterone, but perhaps even more so, from its ability to affect hormone synthesis in brain areas where the *de novo* synthesis of sex steroid hormones, especially estrogen from circulating testosterone, takes place.

### Organizational and activational effects of testosterone and estradiol

Testosterone and estradiol play important roles in organizing and activating sex differences in neural circuits in vertebrates and several detailed studies have been conducted on this topic in rodent models [e.g. (Wu et al., 2009; Juntti et al., 2010; Gegenhuber et al., 2022)] and birds [e.g. (Adkins, 1979; Balthazart et al., 1986; Balthazart, 1991; Gahr, 2004; Court et al., 2020; Bentz et al., 2021)]. In the ruff we expect that the distinct morph-specific profiles of circulating androgens—and the amount of estradiol that can be synthesized from it—affects the development of neural circuitry that contains cell types that are otherwise typically sexually dimorphic.

As noted above, adult Satellite and Faeder males have low levels of testosterone (Figure 1A) but high levels of androstenedione, compared to Independent males (Küpper et al., 2016; Loveland et al., 2021a). These patterns in circulating androgens emerge already in both sexes as early at 10 days old, where variance in testosterone levels in Independents is three times greater than that of Satellites and Faeders, while the variance in androstenedione is greater in inversion morphs compared to Independents (Giraldo-Deck, 2022). Furthermore, we have also confirmed that it persists into adulthood for females as well, where even though circulating levels of testosterone are low, inversion morphs have even lower levels than Independent females (Figure 1A).

The activation of behavioral repertoires during the breeding season for many bird species is also testosterone and estrogen dependent (Wingfield et al., 1987; Ball and Balthazart, 2004; Heimovics et al., 2018). We have demonstrated that the low levels of testosterone in inversion morph males remain virtually unperturbed even after stimulation with gonadotrophin releasing hormone (GnRH). In contrast, for Independent males who do not have the inversion, a GnRH injection induces a textbook-like robust and transient increase in testosterone (Loveland et al., 2021a). It is unclear still whether inversion morphs are only able to make limited amounts of testosterone or whether testosterone is always immediately back converted to androstenedione, such that we only ever detect the end of the reaction (i.e. high androstenedione). Regardless, this suggests potential morph differences in both organizational and activational effects of steroids on physiology and behavior.

Although estradiol has not been studied yet in the ruff, it is well known that many behavioral effects of testosterone can be abolished if administered along with aromatase inhibitors, demonstrating that aromatization of testosterone is necessary for eliciting aggressive and courtship behaviors (Wingfield et al., 1987; Foidart and Balthazart, 1995; Fusani et al., 2003; Balthazart et al., 2004). The ring dove is a species in which roles for testosterone and estradiol in activating specific behaviors in courtship has been studied extensively. In their courtship, chasing, bowing and nest-soliciting are activated by separate hormones. The first two behaviors are more aggressive and are regulated by testosterone, whereas nest-soliciting is specifically controlled by estradiol (Hutchison, 1970, 1971; Cheng and Lehrman, 1975; Adkins-Regan, 1981). Notably, its source is through the aromatization of circulating testosterone inside brain cells. Therefore, we speculate that something similar may occur in ruffs where the lack of aggression components in Satellite courtship displays is perhaps also due to low levels of circulating testosterone, and non-aggressive courtship behaviors (i.e. squats) are dependent on estradiol (from aromatized testosterone), rather than testosterone alone. Future studies could test this directly and see if aromatase inhibitors decrease the frequency of courtship displays.

At this point, we believe the androgenic differences documented thus far in ruffs are only a partial explanation leading to behavioral differences among morphs, and that estrogenic effects have yet to be explored and fully accounted for. We propose that the inversion affects not only circulating testosterone levels among morphs, but also the *de novo* synthesis of estradiol in the brain and that these two events combined, shape both the development and subsequent activation, of morph-specific behaviors in adulthood. Furthermore, increased *HSD17B2* mRNA expression in the brains of embryos suggests that estradiol may also be continually reduced to its less active form, estrone, adding yet another component that could influence morph-specific behavior.

### Beyond typical sex differences in the brain

Elucidating the mechanisms by which chromosomal re-arrangements allow for the evolution of intrasexual behavioral diversity will require a close study of brain areas containing behaviorally relevant neuron types. As examples, we briefly discuss the value in quantifying sex and morph differences in the distribution of aromatase and vasotocin (AVT) neurons in the ruff, as both are organized by, and sensitive to, circulating testosterone levels (Cornil et al., 2011). Profiling AVT neurons and how they relate to androgen levels has proven useful in the study of alternative mating tactics in fishes (reviewed in Mank, 2022). Thus, characterizing both aromatase and AVT systems in the ruff holds great potential for providing insight into the development of morph differences as well as behavioral plasticity in adulthood.

In our testicular expression study, we showed that in Independents and Satellites aromatase and the inversion gene *SDR42E1* have a positive co-expression relationship, whereas no such pattern is present in Faeders (Loveland et al., 2021b). If morph differences in brain aromatase are also present, this could profoundly affect neural organization. Mapping aromatase neurons in the ruff will help meet two goals: refining areas to investigate neuroanatomical differences among male morphs but also between the sexes. Knowing where aromatase neurons are located and identifying their projections has been proposed as an ideal focus for delineating brain areas that integrate sensory information and control motor output for behavioral differences between the sexes (Spool et al., 2022). Aromatase densities and distributions are sexually dimorphic in quail (Foidart et al., 1994; Foidart and Balthazart, 1995; Carere et al., 2007) and aromatase positive cells in the preoptic area that project to the periaqueductal gray are considered responsible for sex differences in testosterone-sensitivity and its ability to elicit copulatory behavior (Carere et al., 2007). Further, aromatase influences an array of behaviors. For example, aromatase neurons in the ventromedial hypothalamus (VMH) are involved in male-male agonistic behaviors in song-sparrows (Soma et al., 2003; Wacker et al., 2010) and estradiol implants in this same brain area induce copulation solicitation in female quail (Wild and Balthazart, 2013). Additionally, we envision that aromatase mapping will be crucial for being able to study hypothalamic nuclei with greater precision and provide a starting point to study female mate choice, which is fundamental to the evolution of sexually selected traits in the ruff.

The nonapeptide AVT is an evolutionarily conserved peptide that modulates a variety of social behaviors including affiliation, parental care, anxiety, aggression, courtship, and sexual behavior (Goodson, 2008). The AVT system consists of several populations of AVT-producing neurons throughout the basal forebrain and midbrain (De Vries and Panzica, 2006). AVT neuron size, cell density, and fiber innervations can be different between the sexes, though the extent can vary depending on the cell group and species (De Vries and Panzica, 2006). Of particular interest is the AVT cell population of the medial bed nucleus of the stria terminalis (BSTm), which is sexually dimorphic in most species, with males having more cells and denser fiber projections than females (De Vries and Panzica, 2006). This cell group is also strongly regulated by sex steroids in seasonally breeding species (Goodson and Bass, 2001; De Vries and Panzica, 2006; Kelly et al., 2011). The BSTm AVT cell group modulates grouping behavior, affiliation, and aggression in a sex-specific manner in estrildid finches (Kelly et al., 2011), and courtship in chicken (Xie et al., 2011). Additionally, aromatase colocalizes with BSTm AVT neurons in zebra finches (Kabelik et al., 2010a), therefore highlighting the potential not only for sex steroids to shape the anatomy of this cell group (i.e., neuronal densities) but to also rapidly modulate behavior in a sex-specific manner (Kelly and Wilson, 2020). We hypothesize that BSTm AVT cell numbers will differ not only by sex, but also by morph in the ruff given the morph differences in sex steroid profiles. Further, it is feasible that testosterone may regulate BSTm AVT neural activity as has been observed in zebra finches (Kabelik et al., 2010b).

## Conclusion and future directions

In the ruff, a genetic re-arrangement present in both sexes has led to the evolution of alternative mating tactics that showcase male phenotypes with distinct appearance, behavior and hormonal profiles. We offer novel insights on *HSD17B2*, a candidate gene expected to be a major explanatory variable for hormonal differences among morphs. We identified a missense mutation in the *HSD17B2* sequence of Satellite and Faeder inversions that has evidence in its human ortholog to be sufficient to produce a dramatic increase in catalytic activity (Sager et al., 2021). This possible enhancement of catalytic activity along with variety in dimer conformations could both contribute to the high androstenedione and low testosterone hormone profiles of inversion morphs. To add meaningful validation to the *HSD17B2* missense mutation findings, knowledge of functional differences that are actually conferred by inversion morph variants of this gene is crucial. For example, site-directed mutagenesis experiments could directly test the role of the leucine to phenylalanine mutation, all while accounting for dimerization combinations.

Neural circuits that underlie behavioral differences between the sexes and between morphs are influenced by the inversion. The presence of the inversion has a direct effect on genetic architecture and consequences proceed onto the transcriptome, proteome and hormone levels (Figure 2B). Ruff inversions produce a low testosterone phenotype in juveniles and adults of both sexes, compared to Independents (Küpper et al., 2016; Loveland et al., 2021a; Giraldo-Deck, 2022). If this pattern extends to embryos, then the inversion modifies the typical influence of sex chromosomes on brain development and organization likely producing even more complex variation between and within sexes. Variation in testosterone levels necessarily impacts the expression of genes that are testosterone-regulated (i.e. through androgen response elements in promoters). We expect estradiol-regulated genes to be similarly affected, though the degree is largely unknown because estradiol levels in circulation and in the brain have not been studied in detail. Nonetheless, low circulating testosterone could also lead to less available substrate for estradiol synthesis inside brain cells. In the white-throated sparrow, another species with inversion-based morphs, the expression of the estrogen receptor ESR1 in the nucleus taenia has been shown to be key in mediating aggression differences between morphs (Merritt et al., 2020). While in both species inversions affect sex hormone pathways in the ruff the variation among morphs in testosterone levels is far greater than that observed between the white-throated sparrow tan and white morphs (Maney and Küpper, 2022).

Inversion morph embryos express higher levels of *HSD17B2* in the brain (Giraldo-Deck, 2022) and future transcriptomic studies shall detail if this persists in adults. We aim to investigate how this could indirectly affect the neural organization of canonically sexually dimorphic neuron types such as aromatase- and vasotocin-producing neurons. We highlight the value of using the ruff to ask questions about the genetic and neural bases that facilitate the evolution of behavioral diversity, both within and between the sexes. We believe that mechanisms for morph-differentiation form an additional layer to those of sexual differentiation and we look forward to elucidating further mechanistic details in this fascinating species.

## Data Availability

Publicly available sequence datasets from (Küpper et al., 2016) were used to retrieve morph-specific HSD17B2 sequences. Hormone and behavior data is available in Edmond, the Open Research Data Repository of the Max Planck Society, at https://doi.org/10.17617/3.5z and in the Phaidra repository at https://phaidra.univie.ac.at/view/o:1604379.
